# Two novel deletion mutations in β-globin gene cause β-thalassemia trait in two Chinese families

**DOI:** 10.1186/s40246-023-00559-4

**Published:** 2023-12-08

**Authors:** Xiuqin Bao, Danqing Qin, Jicheng Wang, Jing Chen, Cuize Yao, Jie Liang, Kailing Liang, Yixia Wang, Yousheng Wang, Li Du, Aihua Yin

**Affiliations:** 1 Medical Genetic Center, Guangdong Women and Children Hospital, Xingnan Road 521, Guangzhou, 510010 Guangdong People’s Republic of China; 2 Maternal and Children Metabolic-Genetic Key Laboratory, Guangdong Women and Children Hospital, Guangzhou, 510010 Guangdong People’s Republic of China; 3 Thalassemia Diagnosis Center, Guangdong Women and Children Hospital, Guangzhou, 510010 Guangdong People’s Republic of China; 4 Prenatal Diagnosis Center, The Second People’s Hospital of Zhaoqing, Zhaoqing, Guangdong People’s Republic of China; 5 Grassroots Guidance and Collaboration Section, Guangdong Women and Children Hospital, Guangzhou, 510010 Guangdong People’s Republic of China

**Keywords:** β-Thalassemia, Novel mutations, β-Thalassemia trait, Premature termination, Truncated peptide

## Abstract

**Background:**

β-Thalassemia is mainly caused by point mutations in the β-globin gene cluster. With the rapid development of sequencing technic, more and more variants are being discovered.

**Results:**

In this study, we found two novel deletion mutations in two unrelated families, *HBB:* c.180delG (termed β^CD59^) and *HBB:* c.382_402delCAGGCTGCCTATCAGAAAGTG (termed β^CD128-134^) in family A and B, respectively. Both the two novel mutations lead to β-thalassemia trait. However, when compounded with other β^0^-thalassemia, it may behave with β-thalassemia intermedia or β-thalassemia major.

**Conclusion:**

Our study broadens the variants spectral of β-thalassemia in Chinese population and provides theoretical guidance for the prenatal diagnosis.

**Supplementary Information:**

The online version contains supplementary material available at 10.1186/s40246-023-00559-4.

## Introduction

β-Thalassemia is mainly caused by point mutations in *HBB* gene and results in reduced (β^+^) or absent (β^0^) of β-globin chains of hemoglobin [1]. It includes three main forms: thalassemia major, intermedia and minor. Nowadays, more than 950 variants in *HBB* gene have been found (HbVar database, http://globin.bx.psu.edu), among which β^CD41-42^ (*HBB*: c.126_127delCTTT, β^0^ thalassemia) and β^IVS-II-654^ (*HBB*: c.316-197C > T, β^+^ thalassemia) were the main genotypes in southern China [2, 3]. The reduction or absence of β-globin chains depends on the variants that occur. Variants in *HBB* coding region, including nonsense mutation, start codon mutation and frame shift, usually affect the translation of *HBB* and lead to β^0^- thalassemia. Here we found two novel mutations, *HBB:* c.180delG (termed β^CD59^) and *HBB:* c.382_402delCAGGCTGCCTATCAGAAAGTG (termed β^CD128-134^) in two Chinese families. β^CD59^ mutation caused frame shift and premature termination of the encoded peptide, while β^CD128-134^ mutation resulted in truncated peptide.

## Materials and methods

### Hematological analysis

Peripheral blood (PB) samples were collected to determine the hematological parameters by using a Sysmex XN5000 automated hematology analyzer (Sysmex Corporation, Kobe, Japan). Hb quantification was performed by automated capillary electrophoresis system (CE) (Sebia Capillarys 2, France). The data are shown in Additional file 1: Table S1. All subjects provided written informed consent.

### Thalassemia variants detection

The common 3 types of α-thalassemia mutations [− α^3.7^ (rightward), − α^4.2^ (leftward), –^SEA^ (Southeast Asian), Hb Constant Spring (Hb CS or *HBA2*: c.427T > C), Hb Quong Sze (Hb QS or *HBA2*: c.377T > C) and Hb Westmead or *HBA2*: c.369C > G] and 17 types of β-thalassemia mutations [codons 41/42 (–TTCT) (*HBB*: c.126_127delCTTT), IVS-II-654 (C > T) (*HBB*: c.316-197C > T) –28 (A > G) (*HBB*: c.-78A > G), codons 71/72 (+ A) (*HBB*: c.216_217insA), codon 17 (*A*AG > *T*AG) (*HBB*: c.52A > T), codon 26 (*G*AG > *A*AG) (Hb E or *HBB*: c.79G > A), codon 31 (–C) (*HBB*: c.94delC), codons 27/28 (+ C) (*HBB*: c.84_85insC), IVS-I-1 (G > T) (*HBB*: c.92 + 1(G > T), codon 43 (*G*AG > *T*AG) (*HBB*: c.130G > T), − 32 (C > A) (*HBB*: c.-82 > A), − 29 (A > G) (*HBB*: c.-79A > G), − 30 (T > C) (*HBB*: c.-80T > C), codons 14/15 (+ G) (*HBB*: c.45_46insG), Cap + 40–43 (–AAACA) (*HBB*: c.-11_-8delAAACA), initiation codon (A*T*G > A*G*G) (*HBB*: c.2T > G) and IVS-I-5 (G > C) (*HBB*: c.92 + 5G > C)] in southern China were detected by using suspension array system as previously reported [4].

### Sanger sequencing

Sanger sequencing was performed to detect the mutation in *HBA1* (MIM 141800), *HBA2*, *HBB* (MIM 141900) and *HBG* (MIM 142200) genes (PCR primers were as follows: *HBA1*: forward primer 5′-TGGAGGGTGGAGACGTCCTG-3′; reverse primer 5′-TCCATCCCCTCCTCCCGCCCCTGCCTTTTC-3′. *HBA2*: forward primer 5′-TGGAGGGTGGAGACGTCCTG-3′; reverse primer 5′-CCATTGTTGGCACATTCCGG-3′; *HBB*: *HBBE1* forward primer 5′-CCAATCTACTCCCAGGAGCAG-3′; reverse primer 5′-TGAGGTTGTCCAGGTGAGC-3′; *HBBE2* forward primer 5′-GATCTGTCCACTCCTGATGC-3′; reverse primer 5′-GGTAGCTGGATTGTAGCTGC-3′; *HBBE3* forward primer 5′-TTCTGGGTTAAGGCAATAGCAA-3′; reverse primer 5′-AGGGGCTGTTGCCAATGTGC-3′; *HBG1*: forward primer 5′-GGCTACTTCATAGGCAGAGT-3′, reverse primer 5′-TACCTTCCCAGGGTTTCTCC-3′; *HBG2*: forward primer 5′-AGCCGCCTAACACTTTGAGCA-3′; reverse primer 5′-TACCTTCCCAGGGTTTCTCC-3′).

## Results

**Fig. 1:**
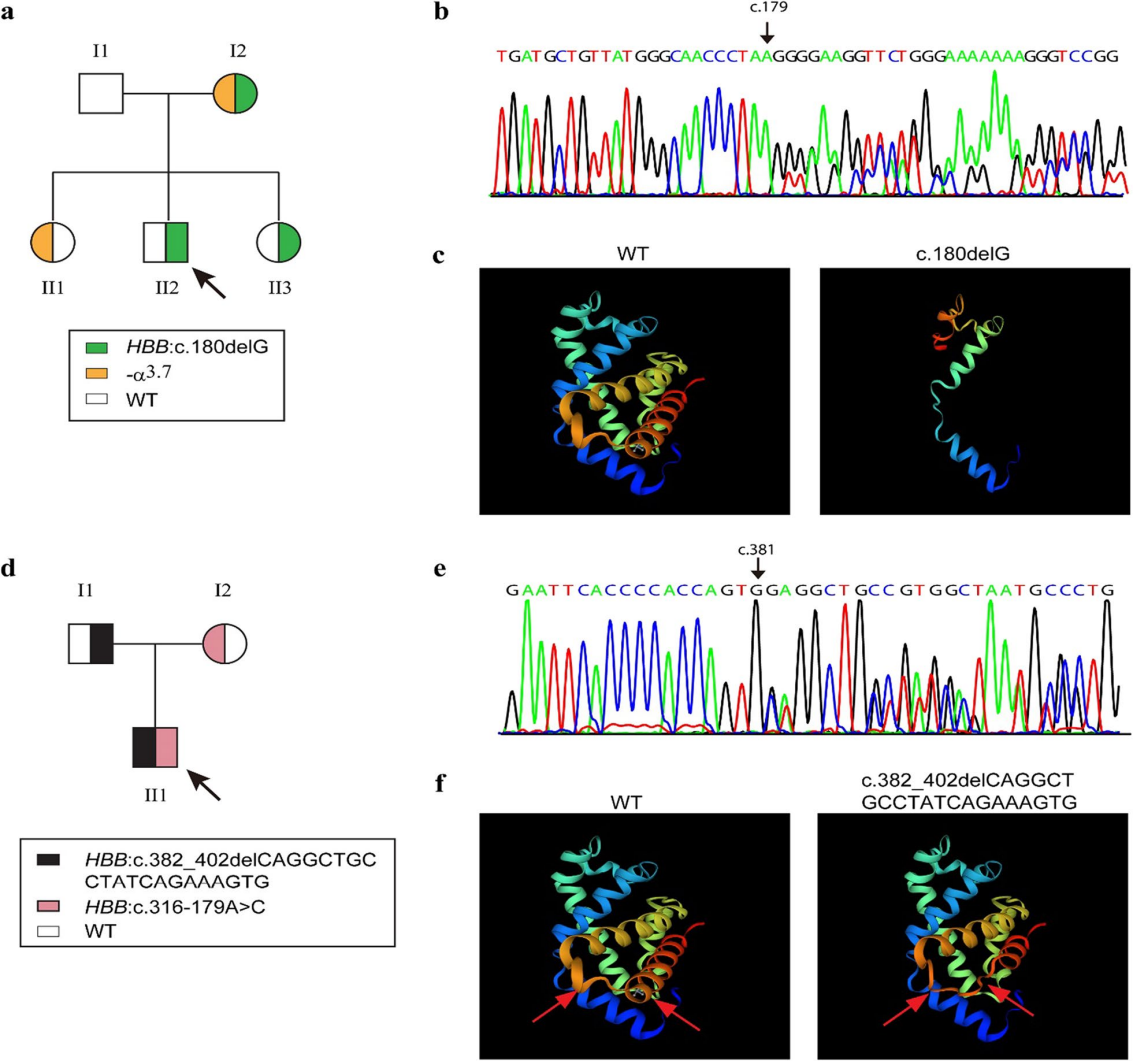
The pedigrees and molecular analysis of the two novel deletion mutations. **a** The pedigree of family A. The proband was labeled with black arrow. **b** Sanger sequencing of the deletion *HBB*: c.180delG in the proband in family A. **c** The β-globin peptide structure predicted by Swiss-Model. WT, wild type. **d** The pedigree of family B. **e** Sanger sequencing of the deletion *HBB*: c.382_402del CAGGCTGCCTATCAGAAAGTG in the proband in family B. **f** The β-globin peptide structure predicted by Swiss-Model. The different domain between WT and the deletions was labeled by red arrow

The proband (II-2) in family A was a 28-year-old man from Zhaoqing, Guangdong Province, China. The hematological parameters showed that he had red blood cell (RBC) morphologic changes with microcytosis and hypochromia. His hemoglobin (Hb) was 126 g/L, mean corpuscular volume (MCV) was 63.4 fl, while mean corpuscular Hb (MCH) was 19.6 pg (Fig. 1a, Additional file 1: Table S1). Hemoglobin analysis demonstrated an increased HbA2 level (5.2%). The result of β-thalassemia gene detection of the 17 types of common mutations in Chinese population [4] was negative. We then performed Sanger sequencing and found a novel heterozygous 1-bp deletion c.180delG (β^CD59^) in codon 59 in HBB gene (Fig. 1b). This novel deletion was inherited from his mother (I-1), who was heterozygote compounded with − α^3.7^ (Fig. 1a). His younger sister (II-3) also carried the deletion, who behaved as β-thalassemia trait with decreased Hb (100 g/L), MCV (66.8 fl) and MCH (20.3 pg) (Fig. 1a, Additional file 1: Table S1). This novel deletion generated stop codon in codon 60 and resulted in premature termination of the peptide. Prediction of the protein structure was performed using SWISS-MODEL [5], and we observed that β^CD59^ can cause truncated β-globin peptide and moderately alter the construction of the peptide (Fig. 1c).

**Fig. 2:**
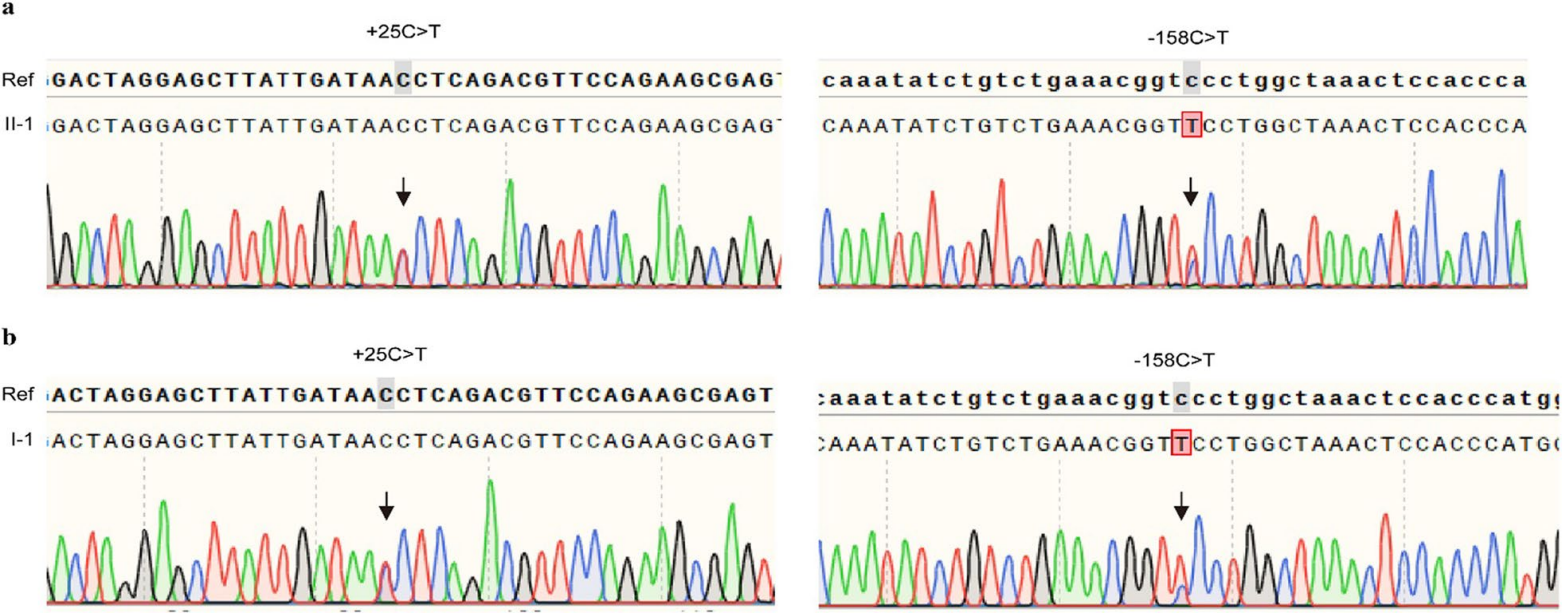
Sanger sequencing analysis of the *HBG* promoter in the proband (**a**) in family B and his father (**b**). The black arrow showed the position of the mutations. *Ref* reference sequence

The proband (II-1) in family B was a 31-year-old man from Guangzhou, Guangdong Province, China. Hemoglobin analysis displayed an increased HbA2 (4.7%) and HbF (7%). He also had microcytosis and hypochromia, in whom Hb was 111 g/L, MCV and MCH was 69.9 fl and 22.8 pg, respectively (Fig. 1d, Additional file 1: Table S1). Genotype result was negative when using suspension array system to detect the 17 types of β-thalassemia mutations. We then performed sanger sequencing and observed a novel 21-bp deletion from 382 to 402 nt of the coding region in *HBB* gene (c.382_402del CAGGCTGCCTATCAGAAAGTG) (Fig. 1e). This deletion was located at the codon 128 to 134 of *HBB* transcript; thus, we termed this mutation β^CD128-134^. We also enrolled his family members and found that this novel deletion was from his father. His father (I-1) also behaved with reduced MCV and MCH (Additional file 1: Table S1). In addition, the proband also carried β^IVS-II-672^ (*HBB:* c.316-179A > C) inherited from his mother (I-2), who behaved normal (Additional file 1: Table S1). To determine whether the 21-bp deletion can influence the construction of the β-globin peptide, we used SWISS-MODE to build the model and ProtParam tool (Expasy ProtParam tool) to analyze the hydropathicity. We found that after deleting the codon 128–134, the β-globin peptide was truncated (Fig. 1f) and the hydropathicity was increased from 0.014 to 0.046. Given that the proband had an increased HbF (7%), we performed Sanger sequencing to detect the hereditary persistence of fetal hemoglobin (HPFH) mutations in *HBG* promoter. We observed that he carried *HBG2*: − 158C > T (NC_000011.9: g.5276169G > A, rs7482144, or *Xmn*I polymorphism) identified to be linked to *HBG1*: + 25G > A (NC_000011.9: g.5271063C > T or rs368698783) [6], which had been reported to regulated the expression of HbF (Fig. 2a). These two mutations were inherited from his father, in whom the HbF level was 0.5% (Fig. 2b).

## Discussion

It has been reported that the point mutation *HBB*: c.[180G > C or 180G > T] [7] caused abnormal hemoglobin Hb J-Lome, with which the heterozygote behaved normal. However, our study found that the deletion of c.180 leads to premature termination of β-globin peptide. In addition, the second deletion mutation, the β^CD128-134^ generated truncated peptide. Mutations occurred in *HBB* coding region that resulting in absent or impaired synthesis of β-globin peptide were defined as β^0^ thalassemia [1, 8]. Therefore, both of these two novel mutations in our study were β^0^ thalassemia, which usually behaved as β-thalassemia trait or β-thalassemia minor, with decreased MCV, MCH and increased HbA2. Heterozygotes of either these two novel mutations compounded with β^0^ or β^+^-thalassemia may lead to β^0^/β^0^ or β^0^/β^+^ thalassemia, which can behave as β-thalassemia major or β-thalassemia intermedia. Unfortunately, we had no recruited such compounded heterozygotes in this study. The proband in family B was compounded heterozygote of β^CD128-134^ and β^IVS-II-672^, in whom the HbF level was elevated (7%), compared with his father (0.5%), who was heterozygote of β^CD128-134^. We detected the HPFH mutations [9] in *HBG* promoter and found he was heterozygote of rs368698783 and rs7482144, two HbF modifiers that can elevate the expression of HbF by demethylating the CpG sites in *HBG* promoter through reducing the enrichment of the repressive transcription factor LRAY [10] and DNA methyltransferase 3 alpha (DNMT3A), as well as protein arginine methyltransferase 5 (PRMT5) [6]. In addition, the heterozygote of β^IVS-II-672^ usually had normal hematological parameters according to the National Center for Biotechnology Information SNP database. Therefore, the SNPs rs368698783 and rs7482144 may explain the increased HbF level in the proband in family B.

## Conclusion

In conclusion, our research found two novel deletion mutations in *HBB* gene, both of which were behaved as β-thalassemia trait or minor. Compounded heterozygote of these two deletions, either β^CD59^ or β^CD128-234^, and β^0^-thalassemia may lead to β-thalassemia major of intermedia. Therefore, our study broadens the spectrum of β-globin variants and provides references for the manifestation of these two novel deletions, especially in the prenatal diagnosis.

### Supplementary Information

**Additional file 1:** The phenotype and genotype data of the probands and their family members.

## Data Availability

All data in this study were available in the figures and tables.
