# Supplementary material for: Two novel deletion mutations in β-globin gene cause β-thalassemia trait in two Chinese families
Source: Hum Genomics. 2023 Dec 8;17:111. doi: 10.1186/s40246-023-00559-4 (PMC10704694; doi:10.1186/s40246-023-00559-4)
Supplement: Supplementary file 1 — Additional file 1: The phenotype and genotype data of the probands and their family members. [file 40246_2023_559_MOESM1_ESM.docx]

**Table S1. the phenotype and genotype data of the probands and their family members.**

|  | Gender (F/M) | Age  (y) | Hb  (g/L) | MCV  (fL) | MCH  (pg) | MCHC  (g/L) | RDW  (%) | HbA  (%) | HbF  (%) | HbA2  (%) | *HBA* | *HBB* |
| --- | --- | --- | --- | --- | --- | --- | --- | --- | --- | --- | --- | --- |
| Family A | | | | | | | | | | | | |
| I-1 | M | 59 | 151 | 88.0 | 29.8 | 339 | 11.8 | 97.1 | 0.0 | 2.9 | αα/αα | β^N^/β^N^ |
| I-2 | F | 60 | 124 | 65.7 | 20.8 | 316 | 14.6 | 94.4 | 0.3 | 5.3 | -α^3.7^/αα | β^CD59^/β^N^ |
| II-1 | F | 34 | 142 | 78.3 | 26.0 | 332 | 13.1 | 97.6 | 0.0 | 2.4 | -α^3.7^/αα | β^N^/β^N^ |
| II-2* | M | 28 | 126 | 63.4 | 19.6 | 309 | 15.5 | 94.8 | 0.0 | 5.2 | αα/αα | β^CD59^/β^N^ |
| II-3 | F | 26 | 100 | 66.8 | 20.2 | 303 | 15.0 | 94.4 | 0.0 | 5.6 | αα/αα | β^CD59^/β^N^ |
| Family B | | | | | | | | | | | | |
| I-1 | M | 59 | 126 | 69.1 | 21.7 | 314 | 14.6 | 94.5 | 0.5 | 5.0 | αα/αα | β^CD128-234^/β^N^ |
| I-2 | F | 55 | 142 | 90.9 | 29.5 | 325 | 12.8 | 97.3 | 0.0 | 2.7 | αα/αα | β^IVS-II-672^/β^N^ |
| II-1* | M | 31 | 111 | 69.9 | 22.8 | 326 | 16.3 | 88.3 | 7.0 | 4.7 | αα/αα | β^CD128-234^/β^IVS-II-672^ |

* Probands.
